# The Prevalence of HIV in Cancer Patients at the Surgical Oncology Unit of Donka University Hospital of Conakry (Guinea)

**DOI:** 10.1155/2015/387896

**Published:** 2015-12-03

**Authors:** Bangaly Traore, Thierno Souleymane Bah, Fode Amara Traore, Mamadou Saliou Sow, Solomana Diane, Mamady Keita, Mohamed Cisse, Moussa Koulibaly, Naby Daouda Camara

**Affiliations:** ^1^Surgical Oncology Unit, Donka University Hospital, Conakry, Guinea; ^2^Infectious and Tropical Disease Department, Donka University Hospital, Conakry, Guinea; ^3^Department of Dermatology and Venereology, Donka University Hospital, Conakry, Guinea; ^4^Department of Pathology, Donka University Hospital, Conakry, Guinea; ^5^Department of General Surgery, University Hospital of Conakry, Guinea

## Abstract

*Aim*. To determine the prevalence of HIV infection among patients seen at the surgical oncology unit of Donka (Conakry, Guinea).* Method*. We conducted a retrospective and descriptive study of HIV infection in cancer patients from May 2007 to December 2012. Social characteristics (age, gender, marital status, and education) and immune status (HIV type, CD4 count) were reviewed.* Results*. Out of 2598 cancer patients, 54 (2.1%) tested positive for HIV. There were 11 (20.4%) defining AIDS and 43 (79.6%) nondefining AIDS cancers. The most frequent cancers were breast (14) (26.0%), non-Hodgkin lymphoma (6) (11.1%), liver (6) (11.1%), eye and annexes (6) (11.1%), and cervical cancer (5) (9.3%). These patients were female in 34 (63.0%) and had a median age of 39 years and body mass index was 20,3 Kg/m^2^. They were unschooled in 40 (74.1%) and married in 35 (64.8%). CD4 count showed a median of 317 cells/mL. Antiretroviral treatment was performed in 40 (74.1%).* Conclusion*. HIV prevalence is higher in patients in our unit of surgical oncology. Breast cancer was the most common in this association. A national survey of a large sample is needed to determine the true prevalence and impact of HIV on cancer prognosis.

## 1. Background

While opportunistic infections continue to be enameled care of patients infected with acquired immunodeficiency, cancer remains overlooked in the care of these patients in developing countries.

According to Center Disease Control (CDC) [1], the revised definition of AIDS criteria includes the biological criteria (CD4 below 200) and/or clinical criteria (opportunistic infections, cancers of the cervix, Kaposi sarcoma, and non-Hodgkin lymphoma) associated with infection by human immunodeficiency virus (HIV).

The occurrence of malignant tumors in patients with HIV infection currently represents for years to come one of the most difficult and controversial challenges of therapeutic management of these patients [2]. As of 2011, 69% of all people infected with HIV live in sub-Saharan Africa. Africa was the epicenter of viral related cancers even prior to the HIV epidemic (e.g., Kaposi's sarcoma (KS), Burkitt's lymphoma, cervical cancer, and hepatocellular carcinoma).

Indeed, 30 to 40% of patients infected with HIV are likely to develop cancer about their disease [3]; the incidence of certain cancers such as Kaposi's sarcoma has decreased since the introduction of potent antiretroviral therapies; other malignancies associated with AIDS do not seem to be affected by the establishment of this new therapeutic strategy [4]. The prevalence coinfection TB/HIV (25.5%) is well known while no study on cancers associated with HIV has been performed in our country [5].

The widespread use of antiretroviral therapy (ARV) in industrialized countries since the mid-1990s led to a dramatic decrease in mortality of patients with HIV infection and incidence of opportunistic infections [6].

However, antiretroviral treatment types HAART also had a significant impact on the natural history of HIV-associated malignancies [7].

A US study found an increase in median survival of AIDS patients from 11.3 months before 1985 to 12.5 months in 1986 to 20.8 months in 1987 for AIDS cases diagnosed in Washington [8]. According to a recent British study, 40% of AIDS patients have cancer during the course of their disease [9].

In France, a study showed that, out of a total of 964 recorded deaths, 149 were attributed to the type of AIDS defining cancers [7].

According to the Regional Office for Africa of WHO, 64% of new cancer cases worldwide are registered in developing countries [10].

Sub-Saharan African countries have experienced nearly 600,000 deaths in 2008 related to tumors. If this epidemiological trend continues due to longer life expectancy and increased exposure to tobacco, Africa will have by 2020 from 800,000 to 1,000,000 new cases of cancer each year [10, 11].

The cancer incidence rate is estimated at 95.5 cases per 10^5^ inhabitants in West Africa and 90.0 per 10^5^ in Guinea [12]. As in most sub-Saharan African countries, the cancer is often diagnosed at an advanced stage of the disease.

There is very little data available on the prevalence of HIV infection in cancer patients in West Africa, especially in our country. It is therefore important to have a first database that will guide future investigations.

This study aims to determine the prevalence of HIV infection among patients seen at the surgical oncology unit of Donka.

## 2. Material and Method

### 2.1. Data

Data from this study were obtained from the hospital and came from surgical oncology unit of Donka national hospital, teaching hospital of Conakry (Guinea).

The study involved the records of cancer patients followed with HIV infection from May 2007 to December 2012.

### 2.2. HIV and Immune Status

The status of HIV infection was determined by the Western blot method. This checkup has become routine for any cancer patient received in our unit since January 2009.

CD4 count was requested for HIV positive patients.

All patients were referred to antiretroviral treatment center for treatment of immune status.

### 2.3. Data Collection

The prevalence has been determined according to the number of patients and pathology subject of consultation or follow-up. Pathologies associated with HIV have been classified into malignant, benign, and inflammatory diseases.

Social characteristics (age, gender, marital status, tobacco and alcohol consumption, education, and body mass index) and immune status (HIV type, CD4 count, and antiretroviral treatment) were reviewed according malignant tumor pathologies, divided into defining AIDS and nondefining AIDS cancers. Gynecological history (age at menarche, first full pregnancy) was determined in women with breast cancer.

### 2.4. Statistical Analysis

This was descriptive and retrospective study. The qualitative variables were presented and analyzed in percentage. The median with interquartile range (IQR) was calculated for the quantitative variables. Defining AIDS and nondefining AIDS cancers were compared in relation to patient age. ANNOVA test was performed and was significant if *p* was less than 0.05.

## 3. Results

Out of 3143 patients received from May 2007 to December 2012, 69 (2.2%) tested positive for HIV in our unit. This proportion was 12 (1.9%) of 625 patients from 2007 to 2008 and 57 (2.3%) of 2518 patients from 2009 to 2012.

Of the 64 HIV-infected patients, 54 (78.3%) had a malignant tumor, 8 (11.6%) of benign and 7 (10.1%) of inflammatory tumors (Table 1).

The prevalence of HIV in cancer patients was 54 on 2598 cases (2.1%). Of the 54 cases of cancers associated with HIV, there were 11 (20.4%) defining AIDS and 43 (79.6%) nondefining AIDS.

These patients were female in 34 (63.0%). Cancer patients had a median age of 39 (IQR 31–48) against 32 (IQR 25–38) for noncancer patients. This median age was different about defining AIDS status: 33 (25.0–40.0) versus 39 (IQR 35.7–50.0) (*p* = 0.007).

Their body mass index was 20,4 Kg/m^2^ (IQR 17.7–21.8). They were unschooled in 40 (74.1%) and married in 35 (64.8%). Tobacco use was found in 13 (24.1%) and alcohol consumption in 14 (20.2%).

All patients were infected with HIV type 1. CD4 count, performed in 22 patients, showed a median of 317 cells/mL (IQR 246.2–534.2). Antiretroviral treatment was performed in 40 (74.1%) patients of which it was performed in 39 only after the cancer diagnosis.

Table 2 shows the distribution of different types of cancers depending on the AIDS definition. Among the cancers defining AIDS, non-Hodgkin's lymphoma (6) (11.1%) and cervical cancer (5) (9.3%) were the most common.

Breast cancer (6) (11.1%), followed by cancer of the liver (6) (11.1%), and eye and annexes (6) (11.1%) were leading nondefining AIDS cancers.

Patients with breast cancer had a median age of 14.0 years (IQR 12.5–15.0) at menarche and 22.0 years (IQR 17.0–25.0) in the first full term pregnancy; and they had 1.0 full pregnancy in term (IQR 0.5–3.0).

## 4. Discussion

This preliminary study shows a high prevalence (2.1%) of the HIV infection in patients with cancer in our surgical oncology unit. This prevalence appeared to be higher than in the general population in Guinea (1.5%) [13] but lower than in the tertiary hospital (6.0%) in Nigeria [14].

Inversely, 30–40% of those HIV-infected patients are at risk of cancer [15]. Risk of cancer occurrence in HIV-infected people is 6.39 times higher compared to uninfected HIV people [16].

In this study, non-AIDS defining cancers were more frequent than defining AIDS cancers (77.8% versus 22.2%). This could be explained partly by the decrease in the number of cancer cases defining AIDS since the beginning of antiretroviral therapy as supported by Yanik et al. in the US [17]. Also, Kaposi's sarcoma (KS) and cervical cancer are mainly managed, respectively, by the dermatology and gynecology departments of teaching hospital. Kaposi's sarcoma is very common in dermatology and the Guinean first data were published by Cisse et al. [18]. In Nigeria, KS and cervical cancer are the most common cancers among non-AIDS defining cancers [19]. Cervical cancer is the most common cancer in Guinea with a high prevalence of human papillomavirus infection [20]. In this study, NHL was leading defining AIDS cancer. This high prevalence of NHL was reported in United States [16].

Breast cancer is the most common of nondefining AIDS cancers. This predominance of breast cancer among nondefining AIDS cancers is reported by studies in sub-Saharan Africa [21, 22]. The HIV infection is very prevalent, about 35.4% among women with breast cancer in Uganda [21] and 19.7% in South Africa [22]. In developed countries, lung cancer is the most common of nondefining AIDS cancers [16, 23].

We noted a female predominance and patients were younger. However, cancer patients were older than those who did not have cancer. Conversely, those with AIDS classifying cancer were younger than those who had a non-AIDS defining illness cancer. The female gender and the young age of patients are already reported by several authors [14, 21]. Unschooling for most patients shows that they are from families with limited resources.

This study has some limitations related to the CD4 count and ART coverage. This could be related to the irregular availability of reagents for CD4 and antiretroviral despite their free access in our country. CD4 count, carried out for 22 cases out of 54, showed no difference in the depth of immunosuppression that cancer is either AIDS defining or not. For defining AIDS cancers, there is no doubt that the risk is higher in the case of lower CD4 count (<200 cells/mL) [24, 25]. However, there are controversies with regard to the depth of immunosuppression in nondefining AIDS cancer [22, 23, 25]. We consider that the ART coverage rate (74.1%) is low in this category of the population infected with HIV, although this rate is higher than the rate of coverage in Guinea (50.0%) [26]. These patients are faced with two problems: HIV/AIDS with the supported opportunistic infections and cancer that is not supported by the national program against AIDS.

That is why a national prospective study is needed to determine the true prevalence of cancer among people living with HIV. It will also be important to determine whether HIV changes the risk factors, clinicopathological futures, and prognosis of these cancers.

## 5. Conclusion

HIV prevalence is higher in patients in our unit of surgical oncology. Breast cancer is the most common in this association. A national survey of a large sample is needed to determine the true prevalence and impact of HIV on cancer prognosis.

## Figures and Tables

**Table 1:** Distribution of HIV patients according to associated diseases.

Primary sites	Associated diseases			
	Malignant tumors	Benign tumors	Inflammatory tumors	All
	*n* (%)	*n* (%)	*n* (%)	*n* (%)
Breast	14 (20.3)	2 (2.9)	1 (1.4)	17 (24.6)
Node	6 (8.7)^*∗*^	2 (2.9)	4 (5.8)	12 (17.4)
Liver	6 (8.7)	—	—	6 (8.7)
Cervix uteri	5 (7.2)	1 (1.4)	2 (2.9)	8 (11.5)
Eyes	4 (5.8)	2 (2.8)	—	5 (7.2)
Skin	4 (5.8)	—	—	4 (5.8)
Soft tissues	3 (4.3)	—	—	3 (4.3)
Eyelids	2 (2.9)	—	—	2 (2.9)
Lung	2 (2.9)	—	—	2 (2.9)
Colon	2 (2.9)	—	—	2 (2.9)
Pancreas	1 (1.4)	—	—	1 (1.4)
Bone	1 (1.4)	—	—	1 (1.4)
Bladder	1 (1.4)	—	—	1 (1.4)
Larynx	1 (1.4)	—	—	1 (1.4)
Kidney	—	1 (1.4)	—	1 (1.4)
Unknown	2 (2.8)	—	—	2 (2.9)
All	**54 (78.3)**	**8 (11.6)**	**7 (10.1)**	**69 (100)**

^*∗*^Non-Hodgkin lymphoma.

**Table 2:** Distribution of cancer type to the SOU during the study period.

Cancer types	Number	%
Defining AIDS		
Non-Hodgkin lymphoma	6	11,1
Cervical cancer	5	9,3
Kaposi sarcoma	1	1,9
Nondefining AIDS		
Breast	14	25,9
Liver	6	11,1
Eyes and adnexa	6	11,1
Skin (carcinoma and melanoma)	4	7,4
Soft tissues	2	3,7
Lung	2	3,7
Colon	2	3,7
Bladder	1	1,9
Pancreas	1	1,9
Bone	1	1,9
Ovary	1	1,9
Larynx	1	1,9
Unknown	1	1,9
All	54	100
